# Supplementary material for: Direct observation of nanoscale dynamics of ferroelectric degradation
Source: Nat Commun. 2021 Apr 7;12:2095. doi: 10.1038/s41467-021-22355-1 (PMC8027400; doi:10.1038/s41467-021-22355-1)
Supplement: Supplementary file 1 — Supplementary Information [file 41467_2021_22355_MOESM1_ESM.pdf]

Supplementary Information

Direct observation of nanoscale dynamics of ferroelectric degradation

Huang et al.

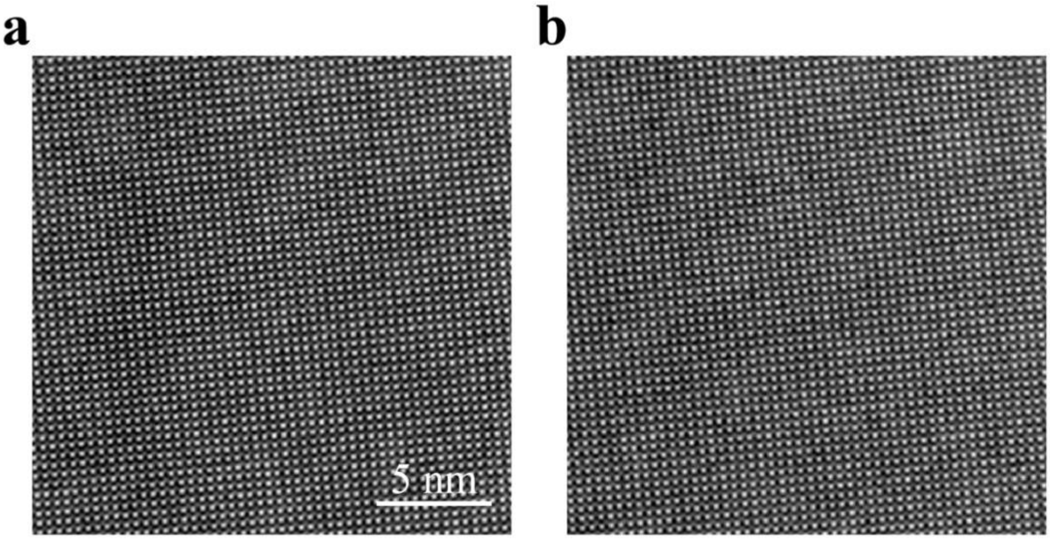

Supplementary Fig. 1 Atomic-resolution STEM-HAADF images of a sample area (**a**) before and (**b**) after 50 cycles of electric loadings without any adjustment of sample tilt and focus. It is clear that the cyclic electric loading did not change the STEM-HAADF image. Because atomic-resolution STEM-HAADF images are sensitive to sample focus and tilt, the almost identical images in **a** and **b** confirm that the system is stable and that the in-situ experiments did not change the focus and tilt.

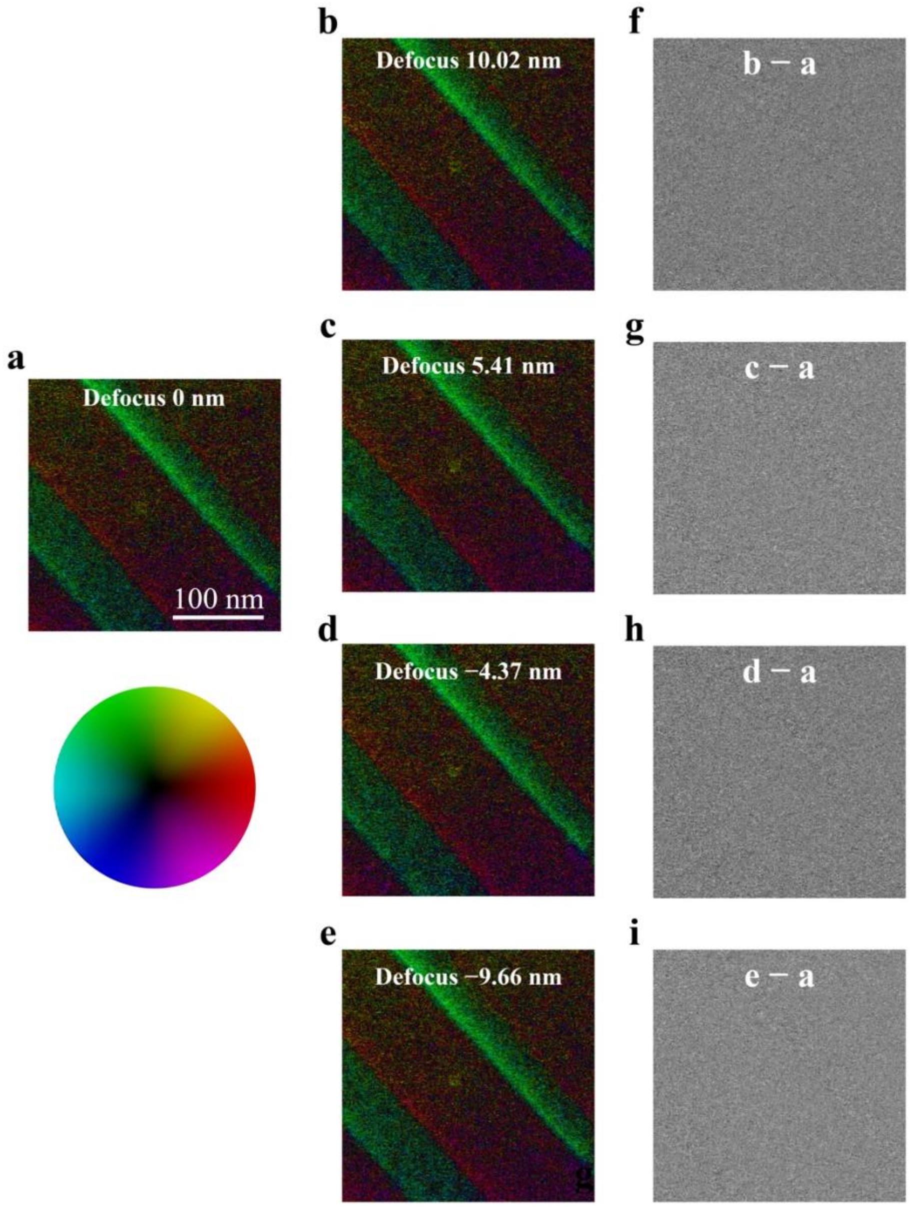

Supplementary Fig. 2 Electric field maps taken at defocus of 0 nm (**a**), 10.02 nm (**b**), 5.41 nm (**c**), −4.37 nm (**d**) and −9.66 nm (**e**). The direction and amplitude of local electric fields are represented by colors, see the color wheel at the bottom of **a**, and image intensity, respectively. Grayscale images **f**, **g**, **h** and **i** were obtained by subtracting the image **a** from the images **b**, **c**, **d** and **e**, respectively. Comparison of **b**, **c**, **d** and **e** to **a** shows no difference in the intensity, as shown in **f**, **g**, **h** and **i**, respectively, indicating that defocus condition does not change the electron field maps observed from STEM-DPC images within a certain defocus range.

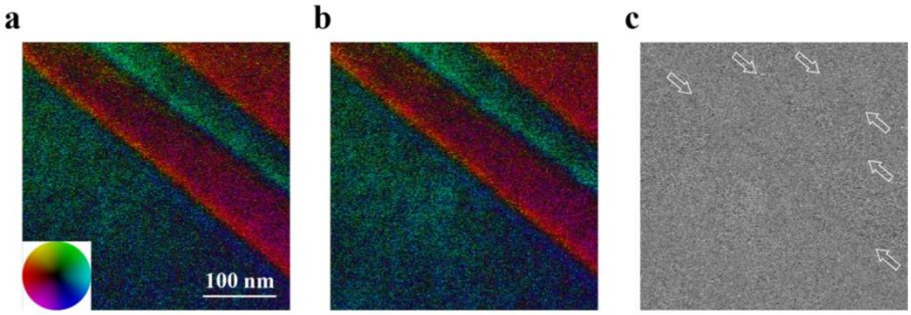

Supplementary Fig. 3 The stability of STEM-DPC images. **a** and **b** Two electric field maps before cyclic electric loading. The direction and amplitude of local electric fields are represented by colors, see the color wheel at the lower left corner of **a**, and image intensity, respectively. **c** A grayscale image obtained from the subtraction of the image in **a** from the image in **b**. While contrast variation in areas marked by the white hollow arrows is easily seen in Fig. 3c, contrast variation in the same areas is hardly identified here, indicating no obvious contrast variation when there is little change in local charge density.

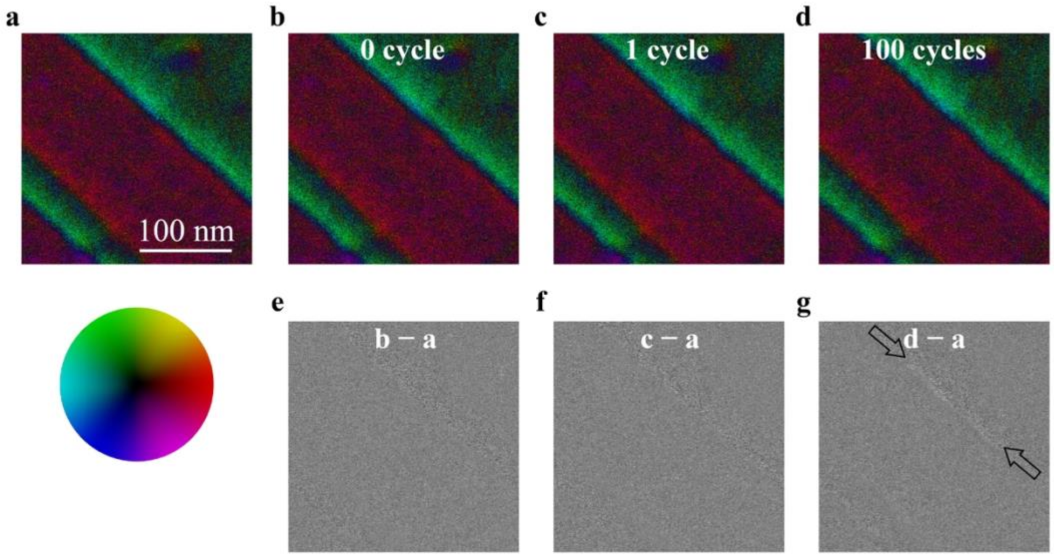

Supplementary Fig. 4 A series of STEM-DPC images with different time and different numbers of electric loading cycles. Electric field maps taken at 0 minute (**a**), 8 minutes later (**b**), after 1 cycle (**c**) and after 100 cycles (**d**) of cyclic electric loadings. The direction and amplitude of local electric fields are represented by colors, see the color wheel at the bottom of **a**, and image intensity, respectively. Grayscale images **e**, **f** and **g** were obtained by subtracting the image **a** from the images **b**, **c** and **d**, respectively. There is no contrast change between **a** and **b**, indicating the stability of STEM-DPC. Also, there is no contrast change between **a** and **c**, indicating little charge accumulation after only one cycle. The black hollow arrows in **g** mark an area with clear intensity variation, indicating charge accumulation there as a result of many cycles of electric loadings. It is worth noting that the domain walls possess the capability of gathering charges, but this capability varies for each domain wall depending on many factors including sample loading history. This can be seen from **g** that the upper right domain shows distinct change in intensity after cyclic electric loading, while the intensity change of the other two domain walls at the bottom left part is not as obvious.

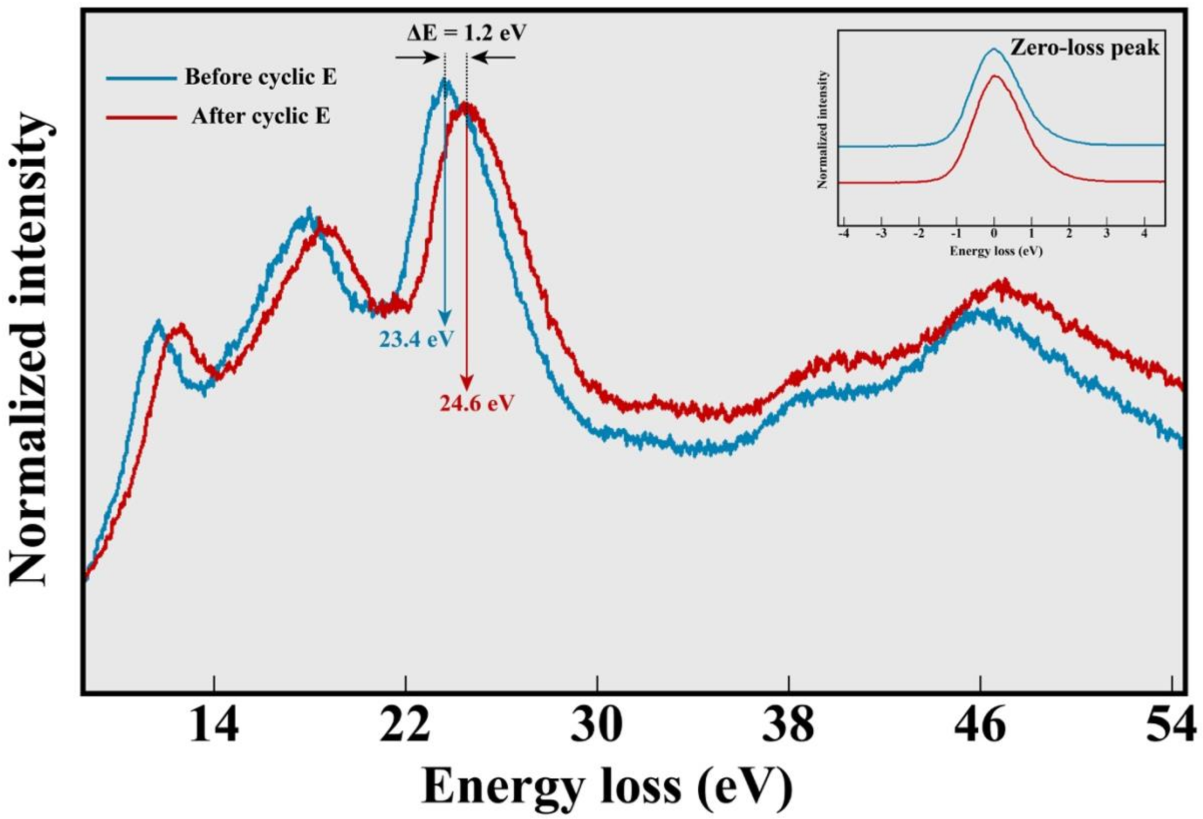

Supplementary Fig. 5 EELS spectra at a domain wall before (blue) and after (red) 100 cycles of electric loading. The spectra were acquired using the dual-EELS mode, at 300 kV with a semi-convergence angle of 17.9 mrad, a collection angle range of 48 – 200 mrad and a beam current of 40 pA. The dual-EELS technique acquires both the zero-loss and the low-loss/core-loss signals, guaranteeing the accuracy of peak shift determination[1]. The energy resolution of the EELS is 1.0 eV, which was measured from the full width at half maximum of the zero-loss peak with an energy dispersion of 0.025 eV/channel. Considering that the energy lost to generate a plasmon is typically in the range 5-25 eV[2] and that the EELS with a higher energy loss can minimize the influences from other factors[3], the energy loss between 20 eV to 25 eV is selected for analysis. The plasma peak shifted 1.2 eV after cyclic electric loading, indicating a change in charge density at the domain wall. This confirms our DPC results in the main text.

Supplementary note 1

Change in charge density at domain walls before and after cyclic electric loadings

According to the plasma theory, change in charge density ΔN can be expressed as[3]

$$\Delta N = \frac{\epsilon_0 m}{e^2} (\omega_{after}^2 - \omega_{before}^2) \tag{1}$$

where ω_{before} and ω_{after} are plasma frequencies before and after cyclic electric loadings, respectively, ϵ_0 is the vacuum permittivity, m is the effective electron mass, and e is the elementary charge of an electron. Plasma frequency can be obtained by the equation[2]

$$\omega_p = \frac{E_p}{\hbar} \tag{2}$$

in which $\hbar$ is the Dirac constant and E_p is plasma energy. Therefore, the change in charge density can be rewritten as

$$\Delta N = \frac{\epsilon_0 m}{e^2 \hbar^2} (E_{after}^2 - E_{before}^2) \tag{3}$$

where E_{before} and E_{after} are the plasma energy before and after cyclic electric loadings, respectively. The change in charge density at domain walls is estimated to be 3.76 electrons/nm^3 when $E_{before} = 23.4$ eV and $E_{after} = 24.6$ eV.

The estimation of the electric field caused by the charge accumulation can be simplified as the electric field between a parallel-plate capacitor using the following equation[4]

$$E = \frac{Q}{A\epsilon} \tag{4}$$

where Q is the amount of charges, A is the area to enclose the charge on the plate and ϵ is the permittivity of PMN-0.38PT. Q can be further expressed as

$$Q = \Delta NeV \tag{5}$$

in which V is the volume of the charges. Therefore, the electric field caused by the charge accumulation can be written as

$$E = \frac{\Delta N e d}{\epsilon} \quad (6)$$

where d is the layer thickness of accumulated charges.

In this simplified calculation, we assume the charges accumulated at the very surface of the sample and a thickness of a unit cell is considered. The adopted relative permittivity of PMN-0.38PT is 734 according to the literature[5]. The estimated electric field is then 37 MV/m. However, the permittivity of ferroelectric materials is highly dependent on the environment and sample conditions, causing the variation of the magnitude of the electric field up to one order. This electric field caused by accumulated charges is strong enough to maintain the *c* domains (the necessary electric field for domain switching in PMN-0.38PT thin lamella in this work is ~0.9 MV/m).

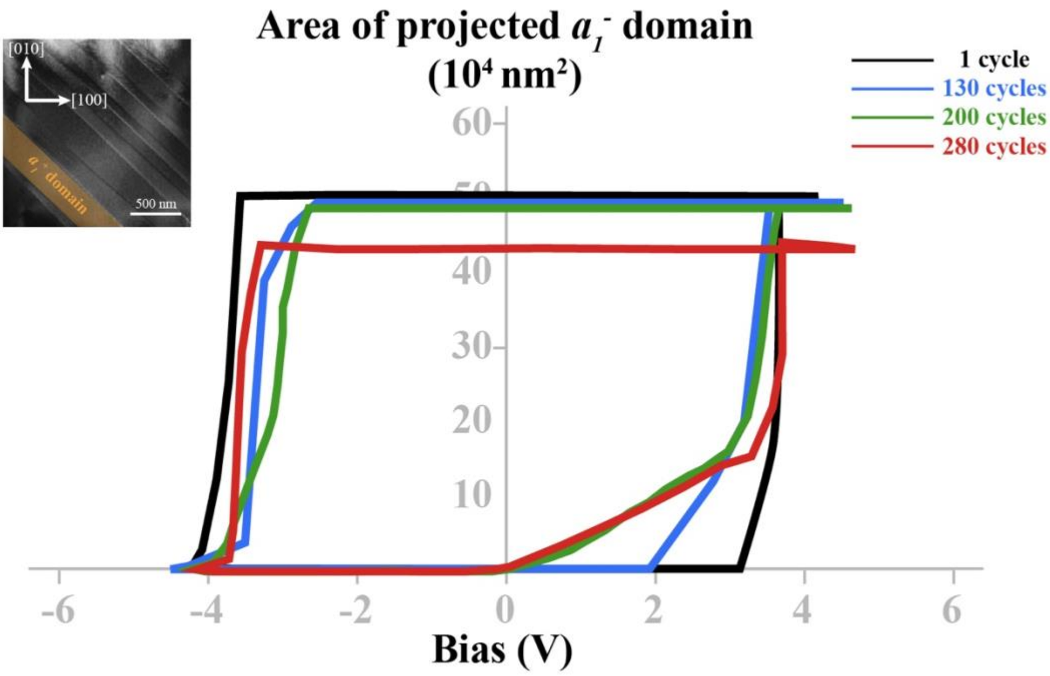

Supplementary Fig. 6 Ferroelectric hysteresis loops of an area of projected a_1^- domain *versus* bias at 1, 130, 200 and 280 cycles. The initial a_1^+ domain marked with yellow shadow in the inset is used for the graph. This graph records the area of the a_1^+ domain switching to a_1^- domain as the applied bias changes at different cycles. All the loops are asymmetric. At the peak positive bias (4.7 V), the switched area decreases as the number of cycles increases, indicating a degradation of ferroelectric property. This is due to the increased number of the frozen domains during the cyclic electrical loading.

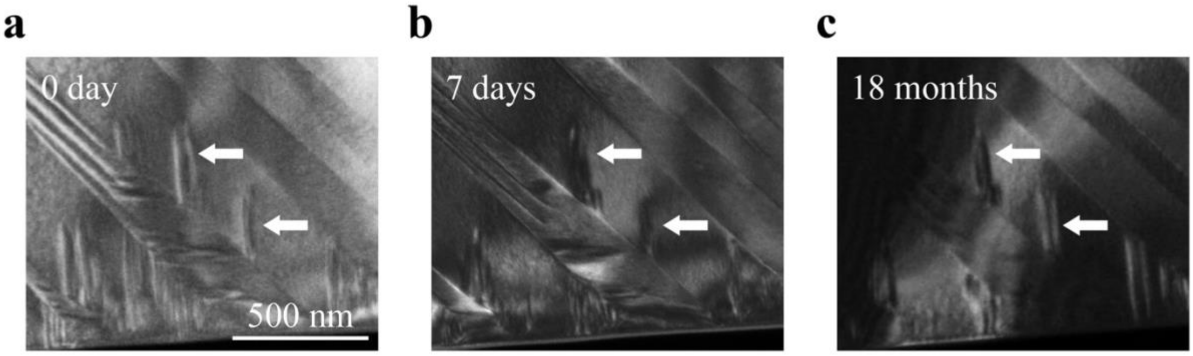

Supplementary Fig. 7 The frozen domains remained 0 day (**a**), 7 days (**b**) and 18 months (**c**) after the cyclic electric loading. The white arrows indicate the frozen domains.

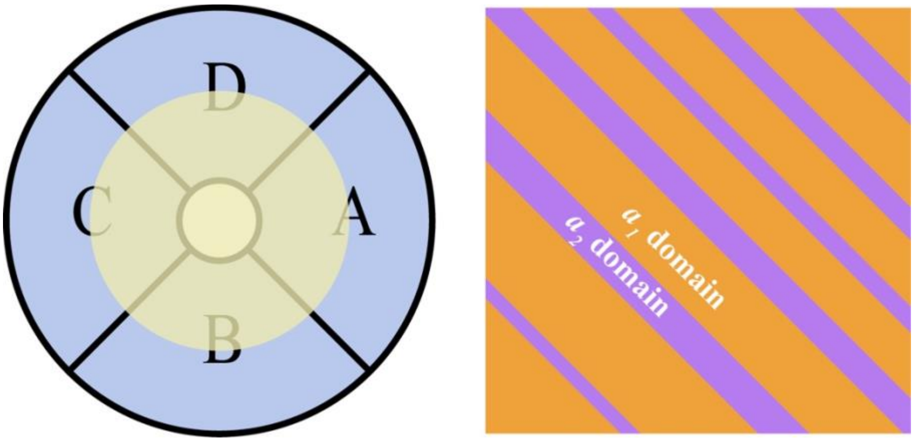

Supplementary Fig. 8 A schematic diagram of the orientation relationship between the detector segments and the sample. The opposite detector segments A and C are aligned in the direction of the electric field. The opposite detector segments B and D are aligned in the direction perpendicular to the electric field. The yellow shadow denotes the bright-field disk.

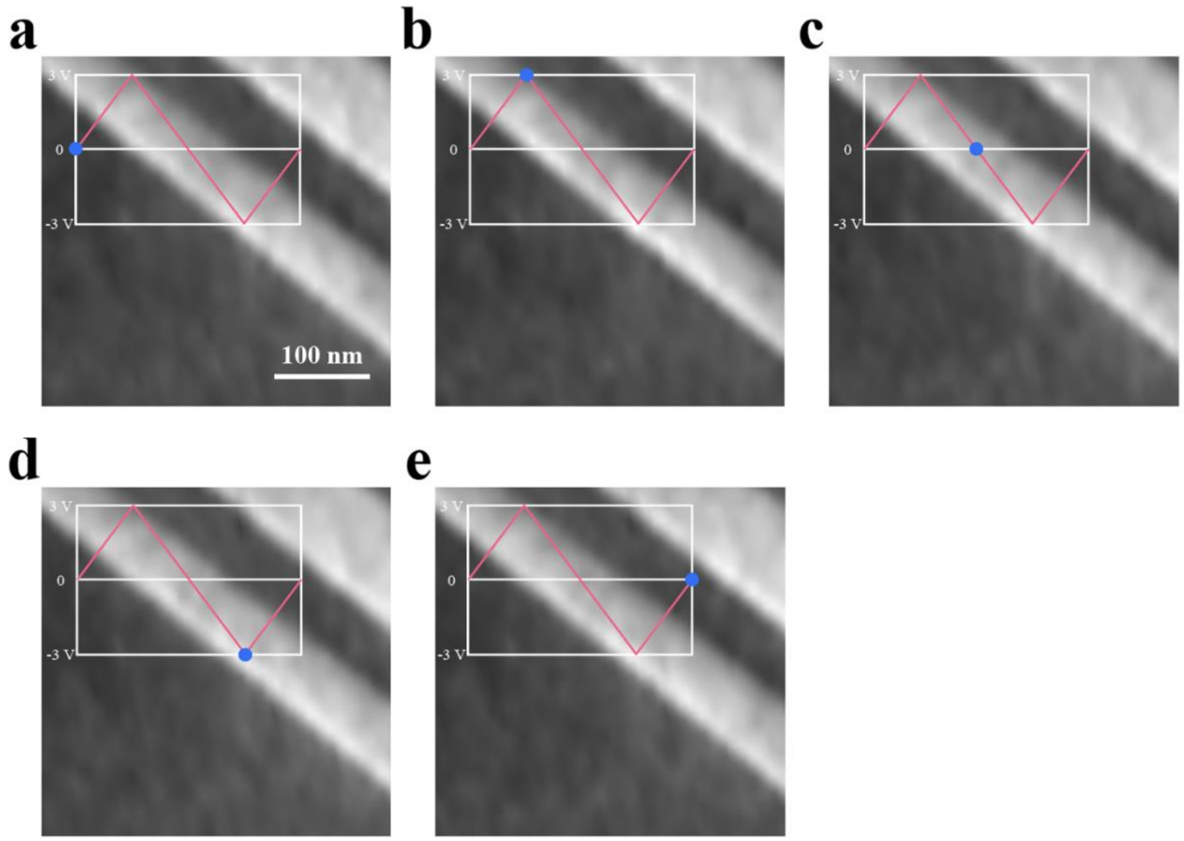

Supplementary Fig. 9 A series of TEM images showing no domain switching occurred in the PMN-0.38PT sample used for STEM-DPC imaging under a cyclic electric loading with a peak voltage of 3.0 V. The blue dot in each image indicates the applied voltage.

References

1. https://eels.info/about/techniques.

2. Williams DB, Carter CB. The transmission electron microscope. In: *Transmission electron microscopy*). Springer (1996).

3. Meng Q, Xu G, Xin H, Stach EA, Zhu Y, Su D. Quantification of charge transfer at the interfaces of oxide thin films. *J Phys Chem A* **123**, 4632-4637 (2019).

4. Griffiths DJ, Inglefield C. Introduction to Electrodynamics, 4th editon.). Cambridge University Press (2017).

5. Sun E, Cao W. Relaxor-based ferroelectric single crystals: Growth, domain engineering, characterization and applications. *Prog Mater Sci* **65**, 124-210 (2014).
