# Supplementary material for: Direct observation of nanoscale dynamics of ferroelectric degradation
Source: Nat Commun. 2021 Apr 7;12:2095. doi: 10.1038/s41467-021-22355-1 (PMC8027400; doi:10.1038/s41467-021-22355-1)
Supplement: Supplementary file 3 — Description of Additional Supplementary Files [file 41467_2021_22355_MOESM3_ESM.pdf]

Description of Additional Supplementary Files

Supplementary Movie 1:

Movie showing the responses of c domains to the electric field stimulation
